# Exploring trends of severe postpartum haemorrhage: a hospital-based study

**DOI:** 10.1186/s12884-023-05702-6

**Published:** 2023-05-19

**Authors:** Silje Pettersen, Ragnhild Sørum Falk, Siri Vangen, Lill Trine Nyfløt

**Affiliations:** 1 Norwegian Research Centre for Women’s Health, Oslo University Hospital, Box 4959, Nydalen, Oslo, 0424 Norway; 2 Institute of Clinical Medicine, University of Oslo, Oslo, Norway; 3 Oslo Centre for Biostatistics and Epidemiology, Oslo University Hospital, Oslo, Norway; 4 Department of Obstetrics, Drammen Hospital, Drammen, Norway

**Keywords:** Postpartum haemorrhage, Trends, Maternal near miss, Blood transfusion, Mode of delivery

## Abstract

**Background:**

Over the past two decades several high-income countries have reported increased rates of postpartum haemorrhage (PPH). Many of the studies are registry studies with limited access to detailed information. We aimed to explore trends of severe PPH in the largest labour ward in Norway during a 10-year period with a hospital based study. Our population constituted all women who gave birth after week 22 at Oslo University Hospital between 2008 and 2017. The main outcome measure was severe PPH, defined as registered blood loss greater than 1500 ml, or transfusion of blood products due to PPH.

**Methods:**

We estimated the incidence of severe PPH and blood transfusions, and performed temporal trend analysis. We performed Poisson regression analysis to investigate associations between pregnancy characteristics and severe PPH, presented using crude incidence rate ratios (IRR) with 95% confidence intervals (CI)s. We also estimated annual percentage change of the linear trends.

**Results:**

Among 96 313 deliveries during the 10-year study period, 2621 (2.7%) were diagnosed with severe PPH. The incidence rate doubled from 17.1/1000 to 2008 to 34.2/1000 in 2017. We also observed an increased rate of women receiving blood transfusion due to PPH, from 12.2/1000 to 2008 to 27.5/1000 in 2017. The rates of invasive procedures to manage severe PPH did not increase, and we did not observe a significant increase in the number of women defined with maternal near miss or massive transfusions. No women died due to PPH during the study period.

**Conclusion:**

We found a significant increasing trend of severe PPH and related blood transfusions during the 10-year study period. We did not find an increase in massive PPH, or in invasive management, and we suspect that the rise can be at least partly explained by increased awareness and early intervention contributing to improved registration of severe PPH.

## Background

Postpartum haemorrhage (PPH) is a major cause of maternal mortality and morbidity, and is estimated to cause 27% of maternal deaths worldwide, and 16% in high-resource countries [1]. Notably, in high-resource countries, overall maternal mortality is low, and deaths due to PPH are rare [2]. However, over the last 20 years, increased PPH incidence rates have been described in several high-income countries [3–5], as well as an increased rate of blood transfusions [6]. Some studies have also demonstrated an increased rate of massive transfusions [7], while a recent Swedish study found a decreasing rate of transfusions of three units or more [8].

The cause of this rise in PPH is unclear [4, 9], although studies have described increasing rates of risk factors, including both maternal characteristics and obstetrical factors [5, 10, 11]. Among the known PPH risk factors, some are related to maternal characteristics (e.g. age, parity, and previous caesarean section), some are introduced in pregnancy (e.g. placenta previa, assisted reproduction, and multiple pregnancies), and others emerge as the pregnancy culminates (e.g. obstetrical interventions, such as induction of labour and operative vaginal delivery) [11–15]. The definition of PPH may also influence the incidence and trends. Comparisons between countries and regions are challenging due to the lack of a global consensus on the definition, variations in accessible prevention and management strategies, and difference in guidelines [16–18]. First-line prevention and management include uterine contractive medication, like oxytocin. Haemorrhage can be further managed using other pharmaceuticals, blood transfusion, and invasive surgical procedures. To explore trends of severe PPH, we must investigate associated risk factors, how PPH is defined, and related management techniques. Knowledge about the rates of severe PPH, case management, and outcomes may help evaluate the quality of obstetric care.

In this study, we aimed to describe the incidence and temporal trends of severe PPH, according to estimated blood loss and units of blood transfusions administered in a hospital setting. Furthermore, we stratified the rates of severe PPH by age, parity, previous caesarean section, and mode of delivery, and estimated the frequency of related invasive management.

## Methods

### Study design, definitions, and inclusion

This hospital-based cohort study included women who delivered at Oslo University Hospital, after gestational week 23 + 0, during a 10-year period from 2008 to 2017. Oslo University Hospital is the largest labour ward in Norway, and consists of both low- and high risk units. It is a referral hospital for foetal congenital malformations and extreme prematurity, as well as for maternal heart diseases. Adjacent hospitals sometimes refer women with suspected placenta accreta spectrum disorders, but Oslo University Hospital is not an official referral hospital for this condition. From the hospital database, we identified women registered with postpartum haemorrhage of 1500 ml or more, or who received a blood transfusion after delivery. This is also the definition of severe PPH used by the Norwegian Medical Birth Register. Women with a registered blood loss of 1500 ml or more were identified from the labour ward´s handwritten protocol book or electronic database. Women with blood transfusion after delivery were identified from a registered code of blood transfusion in the hospital records. To confirm cases and collect variables, the medical records were reviewed by two investigators (LTN and SP). Only women with a confirmed severe PPH, and without obvious plotting errors in the hospital records, were included. We excluded women who received a blood transfusion due to anaemia and had no evidence of severe PPH. Blood loss was estimated and registered in the hospital database by an obstetrician or midwife, either by visual estimation or by using graduated drapes or weighing the cumulative blood loss postdelivery.

Blood transfusions included units of plasma and red blood cells. When six or more units of red blood cells were transfused, plasma and red blood cells were registered separately. During the last six years of the study period, all bags of plasma and red blood cells were registered separately. Maternal near miss was defined as receiving six or more bags of red blood cells, or requiring hysterectomy or embolization of pelvic arteries. To ensure data quality, all hospital records were reviewed, and data entered manually into a database built in EpiData Version 4.4.2.6., by the two main investigators, LTN and SP. After completed registration, we cleaned the database, and checked outliers and categorization errors against the medical records.

The background population consisted of all deliveries at Oslo University Hospital, and included all the cases. Deidentified aggregated combined data on the background population relevant to the analysis were provided by the Norwegian Medical Birth Register.

The main outcome was severe PPH. At the time the study was designed, there was no global consensus on the definition of severe PPH, or on determined outcome in studies of PPH. In 2019, an international Delphi consensus study agreed on a core outcome set for studies of the prevention and treatment of PPH and maternal morbidity [19, 20], which will facilitate comparison between studies in the future. In our study, blood loss was reported in ml after the birth of the baby, and blood transfusions as the number of units (plasma or red blood cells). Other outcomes included invasive management (curettage, intrauterine balloon compression, uterine compression sutures, embolization of pelvic arteries, aortic balloon occlusion, and hysterectomy) and maternal death. Available covariates were age, parity, previous caesarean section, multiple pregnancy, gestational age, and placenta previa.

### Guidelines and simulation training to manage postpartum haemorrhage

The National guideline on PPH were updated in the same year as the study started (2008) and again in 2014. The guideline became more detailed in 2014, and the inaccuracy of visual estimation were underlined. However, there were no recommendations on how to estimate blood loss. Furthermore, tranexamic acid as a prophylaxis in women with an increased risk of PPH and delivered with a caesarean section, a time limit for managing retained placenta, and a specific haemoglobin target for blood transfusions were introduced. Advanced Life Support in Obstetrics trainings, ALSO, was first introduced in Norway in 2003, becoming increasingly popular in the following years. In 2008, Oslo University Hospital started systematic simulation trainings on obstetric complications, including management of postpartum haemorrhage, for all midwives and attending physicians. Although there has been minimal changes to the guidelines in the study period, the awareness of severe PPH probably increased, reflected in a more detailed guideline and systematic simulation trainings.

### Ethical considerations

This retrospective study was granted a waiver of individual informed consent by the South-East Regional Ethics Committee to ensure the inclusion of important groups of women, and because the waiver would not affect the rights and welfare of the included women. The study was also considered beneficial to labouring women. The reference number is 2010/109a. An updated approval was given 2018. We de-identified all information obtained from the women’s medical records prior to statistical analysis, and observed strict confidentiality in all stages of the study. We also had a particular awareness about recognisability in publication of results.

### Statistical analysis

Maternal and obstetric characteristics are presented as frequencies and proportions. The included characteristics were age at delivery, parity, multiple pregnancy, mode of delivery, previous caesarean section, placenta previa, and year of delivery. We estimated the rates of severe PPH per 1000 deliveries for the whole study period, and for each year. The exact Poisson method was used to calculate 95% confidence intervals (CI). Associations with a significance level of ≤ 0.05 were considered statistically significant. We used Stata (version 17) to perform statistical analyses (StataCorp. 2021. *Stata Statistical Software: Release 17*. College Station, TX: StataCorp LLC).

We performed Poisson regression analysis to investigate associations between pregnancy characteristics and severe PPH, presented using crude incidence rate ratios (IRR) with 95% CIs. The estimated annual percentage change was calculated as 100 (e^m^ − 1), where m was the coefficient of the variable ‘year’ when considered as a continuous variable. The 95% CI for yearly change was estimated from the standard error of m, which was obtained from the Poisson regression analysis. Adjustment for age was performed by including age in the model as a categorical variable (< 30, 30–39, 40 + years).

We graphically presented the temporal trends in the incidence of severe PPH, blood transfusions, units of blood products, and maternal near miss. Invasive procedure were graphically visualized according to deliveries with severe PPH. We also graphically presented the rates of severe PPH, stratified and adjusted by age, parity, previous caesarean section, and mode of delivery.

## Results

During the study period (2008–2017), there were 96 313 deliveries at Oslo University Hospital. Almost 50% were nulliparous, increasing from 48 to 51% throughout the period. In the background population, the proportion of women with a previous caesarean section was stable at around 10%, while the proportion of women who delivered by caesarean section decreased from 21 to 18%. Among all deliveries, 2.7% were diagnosed with severe PPH (*n* = 2621). Table 1 presents the maternal and obstetrical characteristics with corresponding rates of severe PPH/1000 deliveries, and crude IRRs. Factors associated with an increased crude risk of severe PPH were maternal age ≥ 40 years, nulliparity, multiple pregnancy, previous caesarean section, operative vaginal delivery, caesarean delivery, and placenta previa.

**Table 1:** Characteristics and rates of severe PPH among women delivering at Oslo University Hospital between 2008–2017

	Severe PPH n (%)	Deliveries^1^ n (%)	Rate of severe PPH per 1000 deliveries (95% CI)	IRR (95% CI)
**Total**	2621	96 313	27.2 (26.2–28.3)	
**Age at delivery²**				
< 30 years	747 (28.5)	29 315 (30.4)	25.5 (23.7-27-4)	1.0 (ref)
30–39 years	1642 (62.6)	61 762 (64.1)	26.6 (25.3–27.9)	1.04 (0.96–1.14)
≥ 40 years	232 (8.9)	5235 (5.5)	44.3 (38.8–50.4)	**1.74 (1.5–2.02)**
**Parity**				
Nulliparous	1569 (59.9)	47 682 (49.5)	32.9 (31.3–34.6)	**1.52 (1.39–1.66)**
Para 1	727 (27.7)	33 549 (34.8)	21.7 (20.1–23.3)	1.0 (ref)
Para 2 +	325 (12.4)	15 082 (15.7)	21.6 (19.3–24.0)	0.99 (0.87–1.13)
**Multiple pregnancy**				
No	2406 (91.8)	94 162 (97.8)	25.6 (24.5-26.59)	1.0 (ref)
Yes	215 (8.2)	2151 (2.2)	10.0 (87.0-114.2)	**3.91 (3.43–4.47)**
**Previous caesarean section**				
No	2282 (87.1)	86 779 (90.1)	26.3 (25.2–27.4)	1.0 (ref)
Yes	339 (12.9)	9534 (9.9)	35.6 (31.9–39.6)	**1.34 (1.2–1.51)**
**Mode of delivery**				
Vaginal delivery	1272 (48.5)	65 548 (68.1)	19.4 (18.4–20.5)	1.0 (ref)
Operative Vaginal delivery	595 (22.7)	11 490 (11.9)	51.8 (47.7–65.1)	**2.71 (2.46–2.99)**
Caesarean section	754 (28.8)	19 275 (20.0)	39.1 (36.4–42.0)	**2.02 (1.85–2.21)**
-elective	214 (8.2%)			
-in-labor	540 (20.6%)			
**Placenta previa**				
No	2503 (95.5)	95 942 (99.6)	25.8 (24.8–26.9)	1.0 (ref)
Yes	118 (4.5)	371 (0.4)	318.1 (263.3-380.9)	**12.2 (9.95–14.9)**
**Year of delivery**				
2008	159	9270	17.1 (14.6–20.0)	1.0 (ref)
2009	200	9444	21.2 (18.3–24.3)	**1.23 (1.00-1.52)**
2010	249	9533	26.1 (23.0-29.6)	**1.52 (1.25–1.86)**
2011	225	9297	24.2 (21.1–27.6)	**1.41 (1.15–1.73)**
2012	277	9848	28.1 (24.9–31.6)	**1.64 (1.35–1.99)**
2013	299	9956	30.3 (26.7–33.6)	**1.75 (1.44–2.12)**
2014	294	9898	29.7 (26.4–33.3)	**1.73 (1.43–2.12)**
2015	271	9695	28.0 (24.7–31.5)	**1.63 (1.34–1.98)**
2016	315	9817	31.8 (28.4–35.5)	**1.87 (1.55–2.26)**
2017	332	9555	34.2 (30.7–38.1)	**2.03 (1.68–2.45)**

Bold text indicates statistically significant results (*P* < 0.05)

PPH, postpartum haemorrhage; CI, confidence interval; IRR, crude incidence rate ratio

^1^Number of deliveries at Oslo University Hospital provided from the Medical Birth Registry of Norway

²One women with missing information from total number of deliveries

**Fig. 1:**
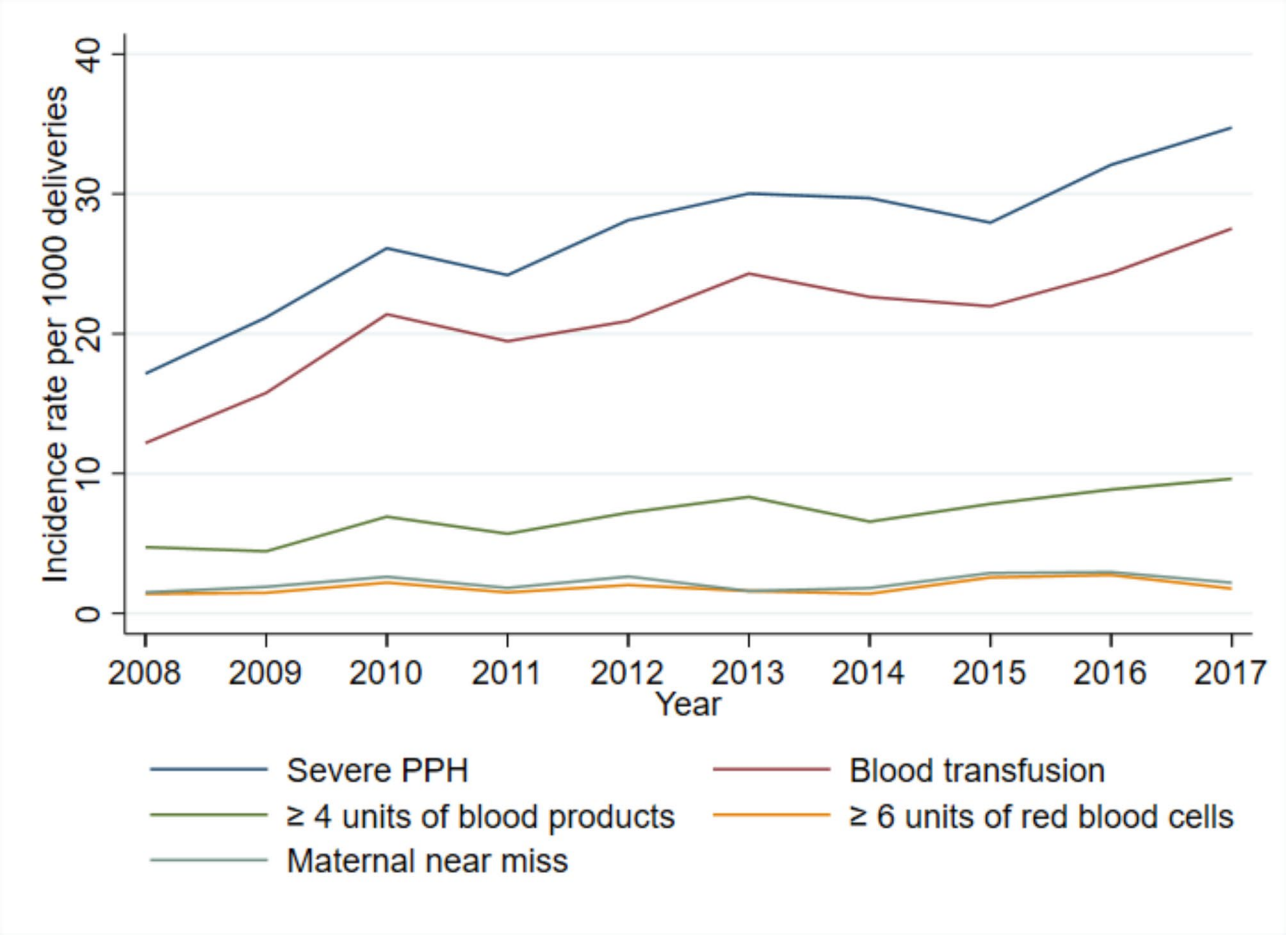
Trends of severe postpartum haemorrhage at Oslo University Hospital (*n* = 96 313 total deliveries)

The incidence of severe PPH among all deliveries at Oslo University Hospital doubled from 17.1/1000 to 2008 to 34.2/1000 in 2017 (Table 1). The trend in incidence of severe PPH was approximately linear (Fig. 1), with an estimated annual percentage change of 6.0% (95% CI 4.6–7.5%, *P* < 0.001). Adjustment for maternal age did not change this estimate (5.9%, 95% CI 4.4–7.4%, *P* < 0.001) (Table 2).

**Table 2:** Rates and estimated annual percentage change of severe PPH at Oslo University Hospital (*n* = 96 313 total deliveries)

	n	Rate per 1000 deliveries (95% CI)	Estimated annual percentage change (95% CI)^1^, P value	Age-adjusted estimated annual percentage change (95% CI)^12^, P value
Severe PPH	2621	27.2 (26.2–28.3)	6.0 (4.6–7.5), < 0.001	5.9 (4.4–7.4), < 0.001
Blood transfusion	2034	21.1 (20.2–22.1)	6.3 (4.7-8.0), < 0.001	6.2 (4.6–7.8), < 0.001
Transfusion ≥ 4 units of blood products	679	7.01 (6.5–7.6)	8.4 (5.5–11.3), < 0.001	8.0 (5.2–10.9), < 0.001
Transfusion ≥ 6 units of red blood cells	181	1.9 (1.6–2.2)	4.5 (0.8–10.0), 0.10	3.9 (1.3–9.4), 0.14
Maternal near miss	211	2.2 (1.9–2.6)	4.6 (0.3–9.7), 0.06	4.0 (0.8–9.1), 0.11

PPH, postpartum haemorrhage; CI, confidence interval

^1^Estimated annual percentage change, assuming a linear trend based on Poisson regression

^2^Adjusted for age by including age in the model (< 30, 30–39, 40 + years)

A total of 2034 women received transfusion of blood products due to PPH (21/1000). The rate of blood product transfusion increased from 12.2/1000 to 2008 to 27.5/1000 in 2017, yielding a yearly increase of 6.3% (95% CI 4.7–8.0%, *P* < 0.001), which was also minimally altered by adjustment for maternal age (Table 2). The incidence of women receiving between one and four units of blood products more than doubled from 9.3/1000 to 2008 to 21.9/1000 in 2017. The number of women receiving four units or more also significantly increased during the ten-year period, with an unadjusted yearly increase of 8.4% (95% CI 5.5–11.3%, *P* < 0.005) (Fig. 1). The incidence of transfusion of six or more units of red blood cells was 1.9/1000 deliveries, and did not significantly increase throughout the study period (4.5%, 95% CI 0.8–10.0%, *P* = 0.10). The highest total number of units administered to one woman was 80 (35 bags of red blood cells and 45 bags of plasma). Among the 2034 women who received a blood transfusion, the mean number of units administered was 3.9. Over half of the women received two units (1168/2034), and almost 14% received four units. Massive transfusions, defined as transfusion of 10 or more units of red blood cells, occurred in 52 women, yielding a rate of 0.54/1000 deliveries, and these cases were evenly distributed throughout the study period.

Maternal near miss occurred in 211 women, yielding a rate of 2.2/1000 deliveries, and a non-significant increase was observed (4.6%, 95% CI 0.3–9.7%, *P* = 0.06). Among the cases defined as maternal near miss, around 50% were nulliparous (105/211), 30% had a history of one or more previous caesarean Sect. (63/211), and 57% delivered by caesarean section in their index pregnancy (120/211). No women died due to PPH during the study period.

**Fig. 2:**
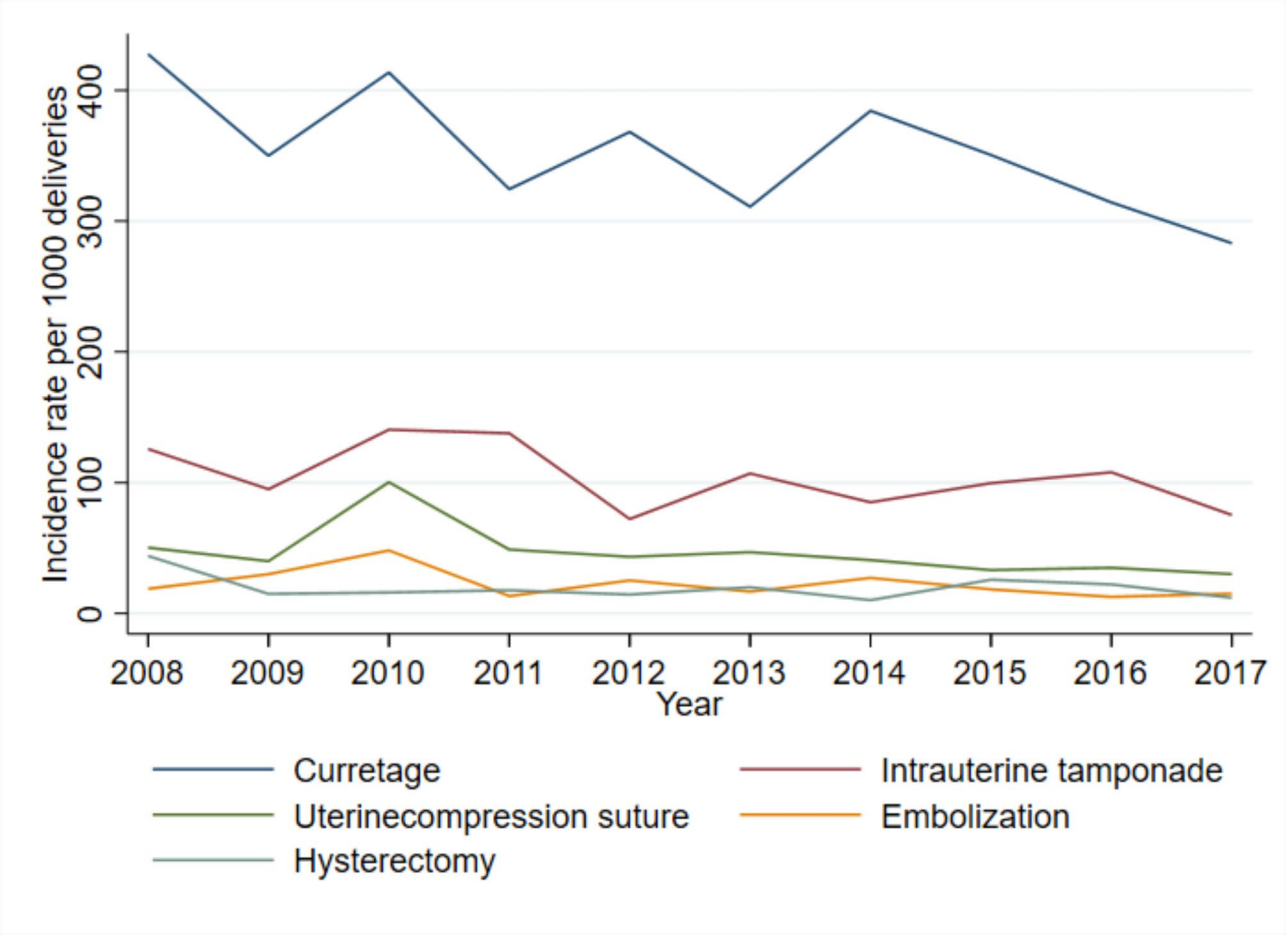
Invasive procedures among women with severe postpartum haemorrhage

Figure 2 presents the year-by-year frequencies of invasive management among women with severe PPH. In 2008, 43% of women with severe PPH underwent a curettage of the uterine cavity, in contrast to 28% in 2017. Intrauterine tamponade, mostly by Bakri balloons, also slightly decreased in frequency during the study period. On the other hand, the rates of uterine compression sutures, embolization of pelvic arteries, and hysterectomies remained stable throughout the time period. Among women with severe PPH, 2.8% (56/2621) underwent embolization of pelvic arteries, yielding a rate of 0.6/1000 deliveries (95% CI 0.46–0.78). The rate of hysterectomy was similar, at 0.51/1000 deliveries (95% CI 0.38–0.67), comprising 1.9% of women with severe PPH (49/2621).

**Fig. 3:**
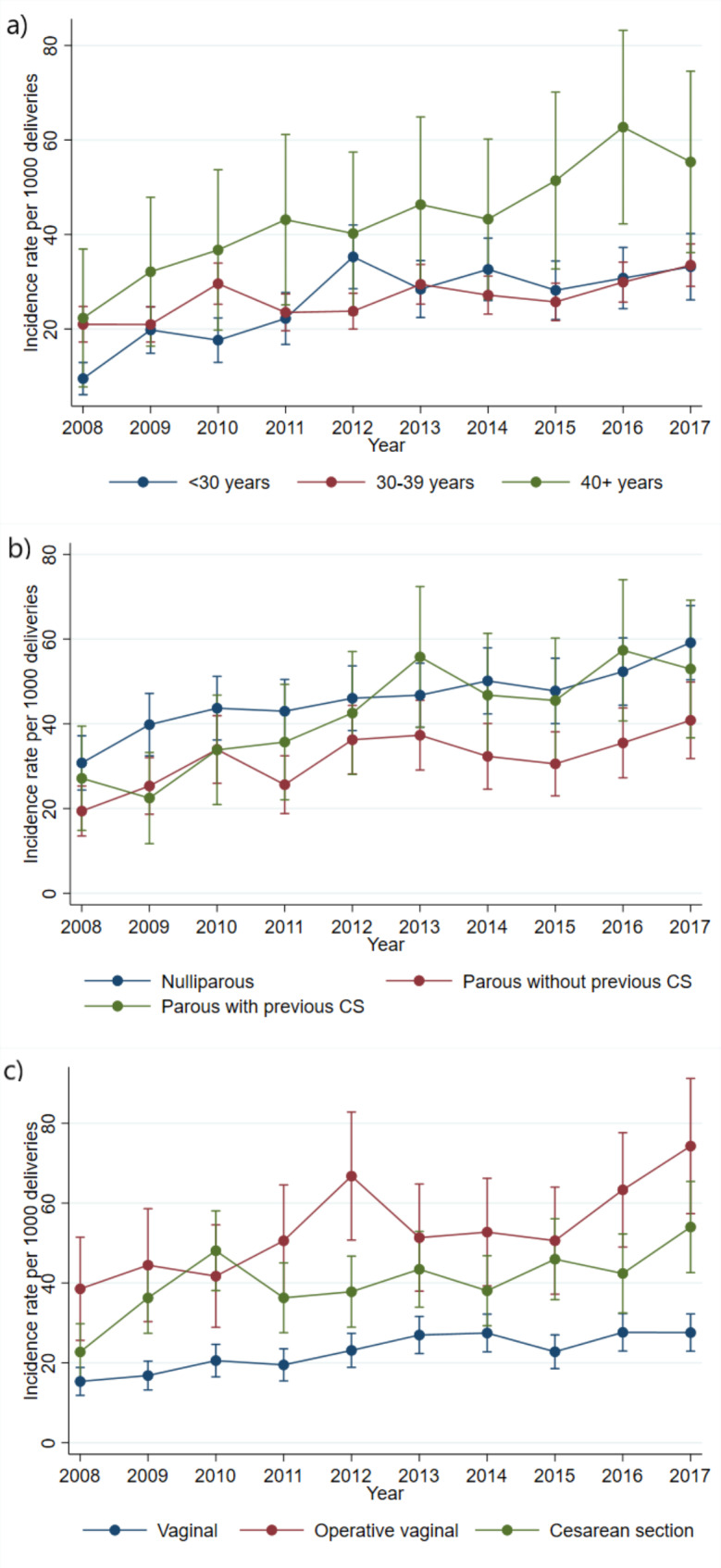
Trends of severe postpartum haemorrhage according to age (**a**), according to parity and previous caesarean section (CS), adjusted for mode of delivery and age (**b**) and according to mode of delivery, adjusted for parity, previous caesarean section and age (**c**)

A higher rate of PPH was associated with maternal age of ≥ 40 years old, compared to women of younger age (< 30 years) (Table 1). When we explored the trend of severe PPH according to maternal age, we observed a significant increase in all age groups (Fig. 3a). The annual increase was higher among women of < 30 years old (9.4%, 95% CI 6.6–12.2%) and among women of ≥ 40 years old (8.5%, 95% CI 3.5–13.6%), compared to women 30–39 years old (4.0%, 95% CI 2.2–5.8%) (Table 3).

**Table 3:** Rates and estimated annual percentage change, stratified and adjusted by maternal age, parity, pervious caesarean section, and mode of delivery

	Severe PPH n (%)	Deliveries^1^ n (%)	Rate of severe PPH per 1000 deliveries (95% CI)	Estimated annual percentage change (95% CI)^3^	P value
**Maternal age**²					
< 30 years	747 (28.5)	29 315 (30.4)	25.5 (23.7–27.4)	9.4 (6.6–12.2)	< 0.001
30–39 years	1642 (62.6)	61 762 (64.1)	26.6 (25.3–27.9)	4.0 (2.2–5.8)	< 0.001
≥ 40 years	232 (8.9)	5235 (5.5)	44.3 (38.8–50.4)	8.5 (3.5–13.6)	< 0.001
**Parity/previous CS**					
Nulliparous	1569 (59.9)	47 682	32.9 (31.3–34.6)	6.1 (4.1–8.2)*	< 0.001
Parous without previous CS	713 (27.2)	39 097	18.2 (16.9–19.6)	5.5 (2.5–8.7)*	< 0.001
Parous with previous CS	339 (12.9)	9534	35.6 (31.9–39.6)	5.2 (2.5–7.9)*	< 0.001
**Mode of delivery**					
Vaginal delivery	1272 (48.5)	65 548 (68.1)	19.4 (18.4–20.5)	5.1 (3.3-7.0)**	< 0.001
Operative vaginal delivery	595 (22.7)	11 490 (11.9)	51.8 (47.7–65.1)	5.8 (3.1–8.5)**	< 0.001
CS	754 (28.8)	19 275 (20.0)	39.1 (36.4–42.0)	8.5 (4.4–12.8)**	< 0.001

PPH, postpartum haemorrhage; CS, caesarean section; CI, confidence interval

^1^Number of deliveries at Oslo University Hospital, provided from the Medical Birth Registry of Norway

²One missing from total number of deliveries

^3^Estimated annual percentage change, assuming a linear trend based on Poisson regression

*Adjusted by age and mode of delivery

**Adjusted by age and parity/previous CS.

In analyses stratified by parity and previous caesarean section, we observed an increasing trend of severe PPH in all subgroups during the study period. These findings were not changed by adjustment for age and mode of delivery (Fig. 3b). The rates increased almost equally, by around 5–6% every year (Table 3).

When stratified according to mode of delivery, the rate of severe PPH exhibited the greatest increase among women who delivered by caesarean section, more than doubling from 21.5/1000 to 53.1/1000 during the study period. Among women with operative vaginal delivery, the rate of severe PPH doubled from 35.8/1000 to 2008 to 71.9 in 2017. The rate of severe PPH after unassisted vaginal delivery increased from 12.9/1000 to 23.9/1000. The increase was adjusted for age, parity, and previous caesarean section (Fig. 3c). The annual increase of severe PPH rate was higher among women with caesarean Sect. (8.5%), than among women with operative vaginal delivery (5.8%), and among women with unassisted deliveries (5.1%) (Table 3).

## Discussion

### Main findings

During the 10-year study period, there was a doubling in the incidence of women diagnosed with severe PPH at Oslo University Hospital. The rate of blood transfusion also doubled during the study period. We did not observe a significant increase in massive transfusions, maternal near miss, or invasive management.

### Strengths and limitations

The main strength of our study was the confirmation of each woman as a case of severe PPH from the hospital database, with detailed information on estimated blood loss and the amount of blood products transfused. This enabled us to exclude women who were falsely registered as experiencing PPH, and to include women who received large amounts of blood transfusions but were not registered with blood loss of ≥ 1500 ml. We also extracted exact information about invasive management, which is often not possible from registry data. Most trend studies are based on birth registers because they can provide large samples, but a hospital-based study will contribute as a supplement to these studies. There is a risk that we missed deliveries that were not registered with a severe PPH, or were not coded with a blood transfusion; however, we believe that we managed to include more cases than would have been included from a registry, while also excluding women who did not have a severe PPH but were registered as such.

The limitations of this study are largely related to the relatively small number of cases, and that it was not a population-based study. Although Oslo University Hospital is the largest labour department in Norway, the population may differ somewhat from the rest of the country, with more urban characteristics, like older parturients and more ethnic diversity. Additionally, since Oslo University Hospital is a referral hospital for the whole country in cases of certain maternal and foetal conditions, it might not be completely generalizable to other areas in a widespread country like Norway. On the other hand, there are no private labour wards in Norway, and all labour wards follow the same national guidelines and provide equal and free healthcare to all pregnant women. Another shortcoming is that we lack information regarding labour induction and augmentation from the last 6 years of the study, and information about body mass index. In these last years, we focused on estimated blood loss, transfusions, and surgical management. The first four years of this study included information on labour induction and augmentation, and the analysis of these data have been presented in a separate paper on risk factors [14]. The estimation of blood loss was done by a combination of visual and metric assessment and represents a limitation in the inclusion of women.

### Interpretation

In this study, we demonstrated an increasing trend of severe PPH and blood transfusion due to PPH during a 10-year period, which is in line with the previously observed rise of PPH in high-income countries [3, 9, 21]. Although this increased trend has yet to be fully explained, increased risk of PPH is reportedly associated with changing maternal factors and increased obstetrical interventions, like caesarean delivery and labour induction [5, 11]. Moreover, while explanatory factors have not yet been clearly identified [4, 22, 23], increased awareness and changing PPH diagnostic practices are suggested to contribute to the rise [4].

An increased rate of blood transfusion due to PPH has also been reported in other studies [6, 21, 24]. Medical record registration of transfusions of blood products are more accurate compared to estimated blood loss due to strict rules on blood transfusions and constitutes a more reliable observation. The increasing rate of blood transfusions may support a true increase in the rate of severe PPH, and in that case, blood transfusions must be considered an adequate life-saving response to severe PPH. On the other hand, the increased rate may also in part be an overuse of an available management. A labour ward is a dynamic place, influenced by traditions and personal opinions, and a shift towards a more liberal use of blood products to manage PPH cannot be excluded, even though the recommendations in the guidelines did not change in the study period. If so, we need a reminder of the possible side-effects of blood transfusions in fertile women, like anaphylactic reactions and potential immunization complications in future pregnancies [25]. Our present analysis did not demonstrate a significant increase in transfusions of more than 6 units of red blood cells. This may be due to the small sample size, but other studies support our finding [26]. No trends of increasing blood transfusion were found in a Dutch study [10], and a Swedish study [8]. Moreover, a French study [6] observed an increase in blood transfusions, but a stable rate of maternal near miss due to PPH. The mean rate of massive transfusions (≥ 10 units of red blood cells) in our study was 0.54/1000 deliveries, which is comparable to that in a Swedish study (0.53/1000 deliveries) [7], but higher than the rate reported from the UK, where 0.23/1000 deliveries involved the administration of eight or more units of red blood cells [27]. We did not estimate the trend of transfusions of ≥ 10 units due to the low number of cases.

The incidence of severe PPH was considerably higher in women who underwent operative vaginal delivery and caesarean section compared to spontaneous vaginal delivery. However, a significant increased rate was present across all modes of delivery. The increase was somewhat higher among women delivered by caesarean section compared to spontaneous and operative vaginal delivery, but with overlapping CI`s. Likewise, a recent study from Sweden analysed the trend of PPH according to the Robson ten group classification [23], and revealed that the rates increased across all modes of delivery, except in prelabour caesarean deliveries. Previous studies have demonstrated that induction of labour and augmentation of labour increases the risk of severe PPH [13, 14]. Medicalization of labour care has escalated over the past decade in Norway [28], and may have played a role in the increasing trend of severe PPH in our population. These interventions may increase the risk in themselves, but the risk may also be increased due to the indications for the intervention, such as prolonged labour, multiple pregnancy, gestational diabetes, estimated large fetus, and maternal age. Unfortunately, we did not have these variables available for our analysis. Studies have reported increased risk of PPH in populations with induction of labour [29] and demonstrated overlapping increased trends of both induction of labour and PPH [28]. On the other hand, a Swedish study that analysed trends of PPH according to Robson groups [23], demonstrated an increasing trend of PPH in women both with and without induction of labour.

The invasive procedures used to manage severe PPH ranged from less-invasive techniques, such as intrauterine balloon tamponade, to highly-invasive procedures, like hysterectomy. The frequencies with which these procedures are used may vary depending on guidelines and availability, as well as hospital traditions and the obstetrician’s experience and preferred method. In our local guideline, a severity trajectory exists for these interventions; PPH is initially managed with a less invasive technique before resorting to more advanced procedures as the haemorrhage increases in severity. As a result, women with severe PPH will often end up with more than one intervention. The cause of PPH and mode of delivery will also have an impact on the sequence of procedures in each case. A French study have demonstrated variations in invasive procedure application between countries [30]. Even within our hospital, traditions and obstetricians’ preferred procedures, may have changed over time and affected our findings, although we do not have the possibility to confirm this. For example, the observed decrease in curettage and intrauterine tamponade among women with severe PPH could reflect a more aggressive and prompt use of uterotonic drugs. However, this may also reflect that severe PPH requiring invasive procedures did not increase, indicating that the most severe cases of PPH did not increase. Likewise, a study from the Netherlands reported an increase in PPH, with a simultaneously decreased incidence of blood transfusions, indicating that only the mildest variants of PPH had increased [10].

In summary, our finding of an increased rate of severe PPH and related blood transfusions is in line with previous reports. However, we did not observe increases in invasive management or maternal near miss and massive transfusions in the same cohort. This may suggest that the rise in severe PPH do not apply to the most severe cases (maternal near miss). One possible explanation could be an increased awareness and enhanced early recognition of PPH, even without any substantial changes in the guidelines. Another possibility is that an improved initial management of PPH has reduced the risk of maternal near miss due to severe PPH.

## Conclusion

Our present study confirmed that the previously reported trend of increasing severe PPH and blood transfusions due to PPH in high-income countries has also occurred in Norway. The increased rate of blood transfusion may indicate that the observed increased rate of severe PPH is true. However, the increase was not associated by similar increases in massive transfusions, maternal near miss, or invasive procedures. Therefore, we may speculate that the rise in severe PPH also is partly due to an improved early recognition and estimation of blood loss in women with severe PPH, simultaneously contributing to enhanced management and prevention of life-threatening haemorrhage.

## Data Availability

The data behind this study are not publicly available because of patient privacy and risk of identifying. Upon reasonable request to the corresponding author it can be made available.

## Abbreviations

CI: Confidence interval
CS: Caesarean section
PPH: Postpartum haemorrhage

## References

[1] Say L, Chou D, Gemmill A, Tuncalp O, Moller AB, Daniels J, Gulmezoglu AM, Temmerman M, Alkema L (2014). Global causes of maternal death: a WHO systematic analysis. The Lancet Global health.

[2] Vangen S, Bodker B, Ellingsen L, Saltvedt S, Gissler M, Geirsson RT, Nyflot LT (2017). Maternal deaths in the nordic countries. Acta Obstet Gynecol Scand.

[3] Knight M, Callaghan WM, Berg C, Alexander S, Bouvier-Colle MH, Ford JB, Joseph KS, Lewis G, Liston RM, Roberts CL (2009). Trends in postpartum hemorrhage in high resource countries: a review and recommendations from the International Postpartum Hemorrhage Collaborative Group. BMC Pregnancy Childbirth.

[4] Mehrabadi A, Liu S, Bartholomew S, Hutcheon JA, Kramer MS, Liston RM, Joseph KS (2014). Temporal trends in postpartum hemorrhage and severe postpartum hemorrhage in Canada from 2003 to 2010. J Obstet Gynaecol Can.

[5] Rossen J, Okland I, Nilsen OB, Eggebo TM (2010). Is there an increase of postpartum hemorrhage, and is severe hemorrhage associated with more frequent use of obstetric interventions?. Acta Obstet Gynecol Scand.

[6] Marocchini M, Lauferon J, Quantin C, Sagot P (2017). Postpartum hemorrhage with transfusion: Trends, near misses, risk factors and management at the scale of a perinatal network. J Gynecol Obstet Hum Reprod.

[7] Thurn L, Wikman A, Westgren M, Lindqvist PG (2019). Massive blood transfusion in relation to delivery: incidence, trends and risk factors: a population-based cohort study. BJOG.

[8] Brynolf A, Zhao J, Wikman A, Öberg S, Sandström A, Edgren G (2021). Patterns of red-cell transfusion use in obstetric practice in sweden 2003–2017: a nationwide study. Vox Sang.

[9] Joseph KS, Rouleau J, Kramer MS, Young DC, Liston RM, Baskett TF (2007). Investigation of an increase in postpartum haemorrhage in Canada. BJOG.

[10] van Stralen G, von Schmidt Auf Altenstadt JF, Bloemenkamp KW, van Roosmalen J, Hukkelhoven CW (2016). Increasing incidence of postpartum hemorrhage: the dutch piece of the puzzle. Acta Obstet Gynecol Scand.

[11] Kramer MS, Dahhou M, Vallerand D, Liston R, Joseph KS (2011). Risk factors for postpartum hemorrhage: can we explain the recent temporal increase?. J Obstet Gynaecol Can.

[12] Kramer MS, Berg C, Abenhaim H, Dahhou M, Rouleau J, Mehrabadi A, Joseph KS (2013). Incidence, risk factors, and temporal trends in severe postpartum hemorrhage. Am J Obstet Gynecol.

[13] Ende HB, Lozada MJ, Chestnut DH, Osmundson SS, Walden RL, Shotwell MS, Bauchat JR (2021). Risk factors for Atonic Postpartum Hemorrhage: a systematic review and Meta-analysis. Obstet Gynecol.

[14] Nyflot LT, Sandven I, Stray-Pedersen B, Pettersen S, Al-Zirqi I, Rosenberg M, Jacobsen AF, Vangen S (2017). Risk factors for severe postpartum hemorrhage: a case-control study. BMC Pregnancy Childbirth.

[15] Al-Zirqi I, Vangen S, Forsen L, Stray-Pedersen B (2008). Prevalence and risk factors of severe obstetric haemorrhage. BJOG.

[16] Borovac-Pinheiro A, Pacagnella RC, Cecatti JG, Miller S, El Ayadi AM, Souza JP, Durocher J, Blumenthal PD, Winikoff B (2018). Postpartum hemorrhage: new insights for definition and diagnosis. Am J Obstet Gynecol.

[17] Dahlke JD, Mendez-Figueroa H, Maggio L, Hauspurg AK, Sperling JD, Chauhan SP, Rouse DJ (2015). Prevention and management of postpartum hemorrhage: a comparison of 4 national guidelines. Am J Obstet Gynecol.

[18] Kerr RS, Weeks AD (2017). Postpartum haemorrhage: a single definition is no longer enough. BJOG.

[19] Meher S, Cuthbert A, Kirkham JJ, Williamson P, Abalos E, Aflaifel N, Bhutta ZA, Bishop A, Blum J, Collins P (2019). Core outcome sets for prevention and treatment of postpartum haemorrhage: an international Delphi consensus study. BJOG.

[20] Schaap T, Bloemenkamp K, Deneux-Tharaux C, Knight M, Langhoff-Roos J, Sullivan E, van den Akker T (2019). Defining definitions: a Delphi study to develop a core outcome set for conditions of severe maternal morbidity. BJOG.

[21] Ford JB, Patterson JA, Seeho SK, Roberts CL (2015). Trends and outcomes of postpartum haemorrhage, 2003–2011. BMC Pregnancy Childbirth.

[22] Ford JB, Roberts CL, Simpson JM, Vaughan J, Cameron CA (2007). Increased postpartum hemorrhage rates in Australia. Int J Gynaecol Obstet.

[23] Ladfors LV, Muraca GM, Zetterqvist J, Butwick A, Stephansson O. Postpartum haemorrhage trends in Sweden using the Robson 10-group classification system: A population-based cohort study. *BJOG* 2021. 10.1111/1471-0528.16931 34536326

[24] Ahmadzia HK, Grotegut CA, James AH (2020). A national update on rates of postpartum haemorrhage and related interventions. Blood Transfus.

[25] Gustavsen A, Njerve IU, Sitras V, Haugen G, Tølløfsrud PA. Akkök Ç A: a young woman with a transfusion-related pregnancy complication. Tidsskr Nor Laegeforen 2020, 140(1). 10.4045/tidsskr.19.0117 31948214

[26] Ramler PI, van den Akker T, Henriquez D, Zwart JJ, van Roosmalen J, van Lith JMM, van der Bom JG (2019). Women receiving massive transfusion due to postpartum hemorrhage: a comparison over time between two nationwide cohort studies. Acta Obstet Gynecol Scand.

[27] Green L, Knight M, Seeney FM, Hopkinson C, Collins PW, Collis RE, Simpson N, Weeks A, Stanworth SS (2016). The epidemiology and outcomes of women with postpartum haemorrhage requiring massive transfusion with eight or more units of red cells: a national cross-sectional study. BJOG.

[28] Haavaldsen C, Morken NH, Saugstad OD, Eskild A (2023). Is the increasing prevalence of labor induction accompanied by changes in pregnancy outcomes? An observational study of all singleton births at gestational weeks 37–42 in Norway during 1999–2019. Acta Obstet Gynecol Scand.

[29] Dahlen HG, Thornton C, Downe S, de Jonge A, Seijmonsbergen-Schermers A, Tracy S, Tracy M, Bisits A, Peters L (2021). Intrapartum interventions and outcomes for women and children following induction of labour at term in uncomplicated pregnancies: a 16-year population-based linked data study. BMJ open.

[30] Kayem G, Dupont C, Bouvier-Colle MH, Rudigoz RC, Deneux-Tharaux C (2016). Invasive therapies for primary postpartum haemorrhage: a population-based study in France. BJOG.

